# Antipsychotics withdrawal in adults with intellectual disability and challenging behaviour: study protocol for a multicentre double-blind placebo-controlled randomised trial

**DOI:** 10.1186/s12888-021-03437-2

**Published:** 2021-09-06

**Authors:** Sylvie Beumer, Pauline Hamers, Alyt Oppewal, Dederieke Maes-Festen

**Affiliations:** 1 Department of General Practice, Chair of Intellectual Disability Medicine, Erasmus MC, University Medical Center Rotterdam, P.O. Box 2040, 3000 CA Rotterdam, the Netherlands; 2 Abrona, Healthcare Organization for People with Intellectual Disability, Huis ter Heide, The Netherlands; 3 Ipse de Bruggen, Healthcare Organization for People with Intellectual Disability, Zoetermeer, The Netherlands

**Keywords:** Intellectual disability, Challenging behaviour, Antipsychotics, Withdrawal, Discontinuation, RCT

## Abstract

**Background:**

In people with intellectual disability (ID) and challenging behaviour, antipsychotics (AP) are often used off-label and for a long period. Despite a lack of evidence for efficacy for challenging behaviour and concerns about common and clinically relevant side effects, complete withdrawal often fails. We postulate three possible hypotheses for withdrawal failure: 1. Influence of subjective interpretation of behavioural symptoms by caregivers and family; 2. Beneficial effects from AP treatment on undiagnosed psychiatric illness, through improvement in sleep or a direct effect on behaviour; and 3. Misinterpretation of withdrawal symptoms as a recurrence of challenging behaviour.

**Methods:**

To investigate our hypotheses, we have designed a multicentre double-blind, placebo-controlled randomised trial in which AP (pipamperone or risperidone) are withdrawn. In the withdrawal group, the AP dose is reduced by 25% every 4 weeks and in the control group the dose remains unaltered. Behaviour, sleep, psychiatric disorders, withdrawal symptoms and side effects will be measured and compared between the two groups. If drop-out from the protocol is similar in both groups (non-inferiority), the first hypothesis will be supported. If drop-out is higher in the withdrawal group and an increase is seen in psychiatric disorders, sleep problems and/or behavioural problems compared to the control group, this suggests effectiveness of AP, and indications for AP use should be reconsidered. If drop-out is higher in the withdrawal group and withdrawal symptoms and side effects are more common in the withdrawal group compared to the control group, this supports the hypothesis that withdrawal symptoms contribute to withdrawal failure.

**Discussion:**

In order to develop AP withdrawal guidelines for people with ID, we need to understand why withdrawal of AP is not successful in the majority of people with ID and challenging behaviour. With this study, we will bridge the gap between the lack of available evidence on AP use and withdrawal on the one hand and the international policy drive to reduce prescription of AP in people with ID and challenging behaviour on the other hand.

**Trial registration:**

This trial is registered in the Netherlands Trial Register (NTR 7232) on October 6, 2018 (www.trialregister.nl).

## Background

### Antipsychotic use in people with intellectual disability and challenging behaviour

Soon after the development of antipsychotics (AP) in the 1950s, they started to be prescribed to people with intellectual disability (ID) [1]. At present, AP use is still high in people with ID. Depending on the heterogeneity of the study population, 14–45% of people with ID use AP [2–8]. Only 22.5% of people with ID have a registered indication such as psychotic disorder or psychotic symptoms [2]. Therefore in the majority of people with ID, AP are prescribed off-label, mostly for challenging behaviour and over long periods [2].

The prevalence of challenging behaviour in people with ID is between 12 and 60%, depending on the definition used and type of cohort [9–11]. The most common presentations of challenging behaviour are aggression, destructive behaviour and self-injurious behaviour [9, 10, 12]. Other presentations are shouting, sexual problem behaviour and pica (an eating disorder with persistent ingestion of non-nutritive substances) [9, 11, 13]. The prevalence of challenging behaviour increases with the severity of the ID [10, 12], and is higher in people with autism or communication and/or social problems [10, 12]. Challenging behaviour can also be related to physical problems, such as pain, visual problems, sleep problems and urinary incontinence [12, 14]. Psychiatric disorders, like depression or anxiety disorder, are common in people with ID, but difficult to recognise [15–17]. Particularly in people with moderate, severe or profound ID, psychiatric disorders might present with more diffuse manifestations of symptoms. This may result in diagnostic overshadowing, as symptoms of psychiatric disorders are falsely attributed to the ID itself [18]. The consequence is that people with ID and a psychiatric disorder will not receive the right treatment, which can result in off-label AP use.

### Effectiveness of antipsychotics for challenging behaviour in people with ID

Despite the long history, almost no research has been done on the (long-term) effectiveness of AP for challenging behaviour in people with ID. Brylewski et al. (2004) concluded in their Cochrane review that there is no evidence from randomised controlled trials that suggests that AP is either helpful or harmful for adults with ID and challenging behaviour [19]. Their review highlighted a lack of good-quality trials [19]. Tyrer et al. (2008) demonstrated that, after 4 weeks of risperidone, haloperidol or placebo use, aggression decreased in people with ID, with the placebo group showing the greatest improvement [20]. Possible causes for this reduction in aggression were a placebo effect, a psychological effect of an external intervention and/or a spontaneous improvement in their behaviour [20].

Jesner et al. (2007) concluded in their Cochrane review that risperidone can be beneficial for some features of autism in people with and without ID, but the available evidence is limited due to the small sample sizes of the three included studies, the lack of a standardised outcome measure allowing comparison of the studies and a lack of long-term follow-up.

One of the possible positive effects that AP might have is a reduction in sleep disturbances, which are common in people with ID [21]. The sedating effect of AP may improve sleep in people with ID and challenging behaviour [22]. Sleep disturbances are associated with problem behaviour in people with ID [23]. Therefore off-label AP for challenging behaviour may in fact be treating (unrecognised) sleep problems.

Despite the lack of evidence for the effectiveness of AP in reducing challenging behaviour, AP are often prescribed for a long period in people with ID. To illustrate, 78% of the participants in the study of De Kuijper et al. (2010) had used AP for more than 10 years [2].

### Relevant side effects of antipsychotics

Although evidence for any effect of the long-term use of AP on challenging behaviour in people with ID is missing, there is convincing evidence that side effects, such as diabetes, metabolic syndrome, extrapyramidal side effects, decreased threshold for seizures, emotional blunting and hyperprolactinemia, are common and clinically relevant [24–27]. The occurrence of side effects is particularly important because this is a vulnerable population with many comorbidities. These side effects are at least partly reversible after withdrawal of AP [25, 28].

Extrapyramidal side effects may frequently be missed in people with ID. Comorbidities such as spasticity, hypertonia, tics or repetitive behaviour make it difficult to distinguish between these comorbidities and extrapyramidal side effects due to AP use. Certain movement disorders, such as akathisia and dyskinesia, can also incorrectly be regarded as challenging behaviour. This can lead to an inadequate treatment with a further increase of the AP dose, while withdrawal is indicated.

### Antipsychotic withdrawal

Despite concerns about side effects and questionable efficacy, the successful withdrawal of AP is not self-evident. In a systematic review, it was found that complete withdrawal from off-label AP was achieved in 4–74% of the people with ID and challenging behaviour, and the proportion of unsuccessful attempts to reduce or discontinue AP was between 0 and 96% [29]. The effects of withdrawal on challenging behaviour are not clear. De Kuijper et al. found that mean ABC (Aberrant Behaviour Checklist) ratings improved significantly for those who achieved complete withdrawal, but baseline ABC scores were significantly lower in people who achieved complete withdrawal versus those who had not achieved complete withdrawal [30]. The wide range in the degree of success of withdrawal could be explained by differences in study designs, heterogeneity of study populations, and methodological shortcomings such as lack of a good description of the intervention, small sample size, selection bias, a lack of blinding of the intervention, no control group or no matched control group and incomplete reporting [29].

We hypothesised three possible reasons for AP withdrawal failure [31]. These hypotheses are as follows:

1. Subjective expectations and interpretations of behavioural symptoms by caregivers, the person with ID and their family have an influence. Their perceptions might be influenced by fear of worsening behaviour after AP withdrawal [32]. Subsequently, this influences the interpretation of behaviour, and the attitudes and apprehensions of caregivers and family with respect to the person with ID. This might contribute to the withdrawal outcome. It has been suggested that successful withdrawal depends, at least in part, on staff and environmental characteristics [33].
2. It cannot be excluded that some people with ID and without a registered indication for AP might benefit from AP treatment. AP may be effective for previously undiagnosed psychiatric illnesses for which AP is indicated [17], possibly due to a lack of (adequate) diagnostic procedures and instruments. In addition, AP may have a beneficial effect on (unrecognised) sleep problems [23]. Furthermore, it cannot be excluded that some people with ID and challenging behaviour (without underlying psychiatric disorders or sleep problems) might benefit from AP treatment.
3. When AP are withdrawn after long-term treatment, withdrawal symptoms such as agitation, mania, akathisia, withdrawal-dyskinesia, anxiety and sleep problems may occur [22, 34]. These symptoms may be misinterpreted as recurrence of the original challenging behaviour, resulting in a request to reinstitute AP treatment.

## Objectives

The aim of the current study is to unravel the mechanism for AP withdrawal failure by testing these three possible hypotheses. We are therefore currently conducting a double-blind, placebo-controlled randomised AP withdrawal trial in people with ID and off-label AP use for challenging behaviour. In this paper, we describe our study protocol.

To investigate the first hypothesis we will compare the percentage of participants completing the protocol in the ‘withdrawal group’ (AP dose is reduced gradually) compared to the control group (AP dose is kept the same). This resembles the clinical practice decision process, where the decision to discontinue AP withdrawal is mostly based on the subjective judgement of professional caregivers, physicians and behavioural scientists. If the failure rate is similar (non-inferiority) in both groups, AP withdrawal failure cannot be caused by AP withdrawal effects and our first hypothesis is supported.

To investigate the second hypothesis, the effects of AP on psychiatric, sleep and behaviour symptoms will be compared in both groups. If AP withdrawal unmasks previously undiagnosed psychiatric disorders or sleep problems or results in increased behavioural problems in the withdrawal group compared to the control group, accompanied by increased drop-out in the withdrawal group, our second hypothesis will be supported.

To investigate the third hypothesis, symptoms commonly associated with withdrawal and side effects will be diagnosed and compared in both groups. If these withdrawal symptoms are more common in the withdrawal group, accompanied by increased drop-out in this group, this supports the third hypothesis that withdrawal symptoms contribute to the failure of AP withdrawal.

## Methods/design

### Setting and design

The study is being conducted within the Academic Collaborative Center ‘Healthy Ageing and Intellectual Disabilities’ (HA-ID). This is a collaboration between three care organisations for people with ID in the Netherlands (Abrona, Amarant and Ipse de Bruggen) and the Intellectual Disability Medicine research group of Erasmus MC, University Medical Center Rotterdam. In order to include enough participants, we are also recruiting participants within other care organisations (Prinsenstichting and Zideris). The care organisations are located in the west, east and south of the Netherlands. They give support to people with borderline to profound ID.

**Fig. 1:**
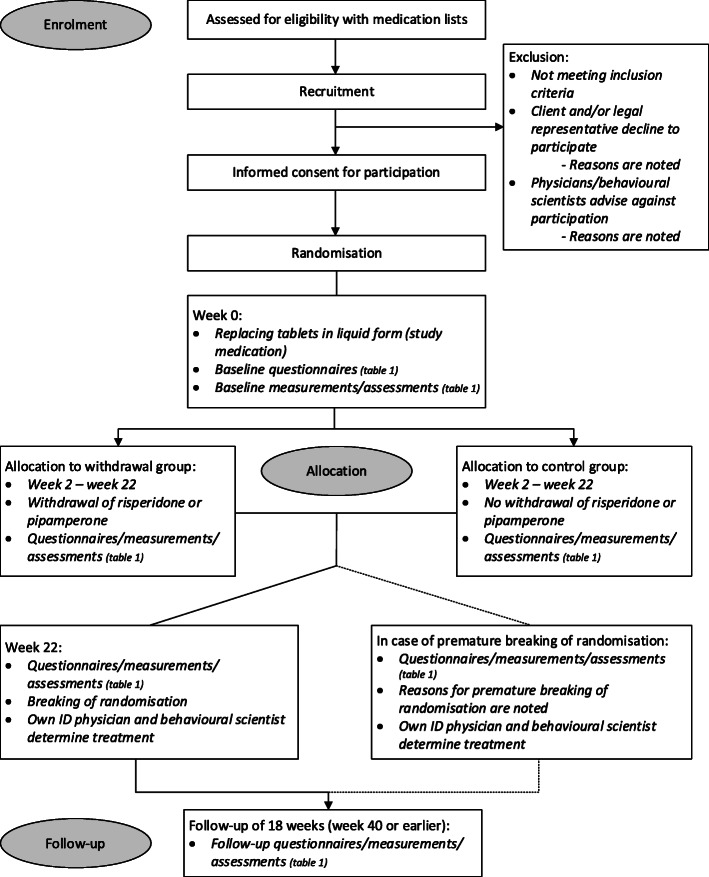
Flow chart of study procedures

To investigate our hypotheses, we designed a multicentre double-blind, placebo-controlled randomised AP withdrawal trial with a non-inferiority design. See Fig. 1 for the flow chart of the study procedures.

### Intervention

**Fig. 2:**
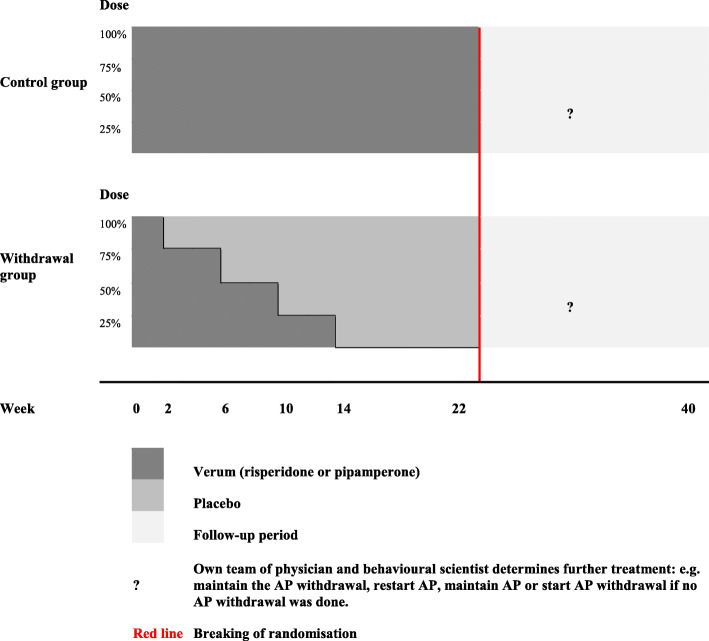
Intervention design

In this study we focus on the withdrawal of risperidone or pipamperone. These AP are the most commonly used AP (over long periods) in people with ID and challenging behaviour in the Netherlands [30]. They are also available in liquid form, enabling gradual dosage adaptation in a placebo-controlled design. For all participants, risperidone or pipamperone tablets will be replaced with medication in a liquid form at the same dose with the same daily administration. If a participant takes the AP more than twice a day, their own physician will be asked to convert it into administration twice a day, in accordance with the prescription policy, before the start of the study. The withdrawal group will have their AP dose reduced by 25% every 4 weeks. In the control group the AP dose will remain unchanged. This is a cautious withdrawal scheme, which has been used in previous studies [30, 33]. We have also opted for this relatively long period between dose reductions to extinguish acute withdrawal symptoms [34]. Dose reduction will start 2 weeks after the participant has switched to the liquid form. The blinding of risperidone/pipamperone or placebo will be broken after 22 weeks. Thereafter, participants will be followed for an open-label period of 18 weeks and they will receive their care as usual. In this follow-up period, the participant’s own team of physicians and behavioural scientists decide on the further treatment: e.g. maintaining the withdrawal, restarting AP, maintaining AP or starting AP withdrawal if no withdrawal was done. The total study duration is 40 weeks (or shorter in case of premature breaking of the randomisation; in other words the duration is equal to ‘week of breaking the randomisation’ + 18 weeks). See Fig. 2 for visualisation of the intervention design.

### Participants

#### Inclusion and exclusion criteria

To be included in this study, participants must be 18 years or older, have an ID (IQ ≤70), have used off-label AP (risperidone or pipamperone) for challenging behaviour for more than 1 year, and live in homes run by the participating ID care organisations. Their own physician(s) and behavioural scientist(s) will be asked to distinguish between challenging behaviour and a psychiatric diagnosis as indication for AP. For this distinction a review of the medical file is necessary if psychotic symptoms or schizophrenia associated with ID were diagnosed in the past. This is an important consideration given the inclusion and exclusion criteria. People are excluded in the case of a current diagnosis of psychosis, psychotic disorder not otherwise specified, dementia, schizophrenia, an active delirium or a delirium in the past month, a failed attempt to withdraw AP in the last 6 months, and/or usage of more than one AP. Participants are allowed to use co-medication or to start co-medication (except for AP) during the study. Medication changes during the study and indications for prescription will be registered.

#### Recruitment and informed consent procedure

The physicians of the care organisations will request a list from the pharmacy of people who are 18 years or older and who use monotherapy risperidone or pipamperone. The physician and behavioural scientist will screen this list against the inclusion and exclusion criteria. The physician and behavioural scientist will decide if the potential participant and/or their legal representative may be approached. Reasons why the physician and behavioural scientist decide not to approach potential participants will be noted anonymously. Individuals who meet the inclusion criteria and may be approached by the physician and behavioural scientist will be asked if they would like to receive the study information. Because not all people with ID are able to give informed consent themselves, their behavioural scientist and/or physician will be asked if the potential participant is able to understand the adapted study information and decide whether to give informed consent for participation. If the potential participant is able to understand the adapted study information and informed consent form, both are sent to the potential participant. If the potential participant is unable to understand the information and unable to give consent for participation, the information and informed consent form will be sent to their legal representative. Also, an information letter will be sent to the professional caregivers of the potential participant by e-mail. If the potential participant and/or their legal representative decline to participate, the reasons why are noted. After permission to participate has been given, participants can withdraw from the study at any time without any consequences.

#### Randomisation and blinding

The trial pharmacist will randomise the participants with the use of a schedule in Excel. This schedule will be created by an independent biomedical statistician. Participants will be randomly assigned to one of two groups: the withdrawal group or the control group. Block stratification with a size of four will be used to ensure that the participants are properly distributed over both groups. Stratification will take place for the following factors: care organisation (to eliminate possible differences between organisations), pervasive developmental disorders (due to the possible effectiveness of AP [35]), and being able to undergo home polysomnography. Participants, care staff, physicians and researchers are blinded for the allocation to the withdrawal or control group. If premature breaking of the randomisation code is necessary, the physician and behavioural scientist can ask the researchers for this information. Also, the participant and/or legal representative can, after consultation with their own physician and behavioural scientist, ask the researchers for premature breaking of the randomisation code. The trial pharmacist is the only person who can break the randomisation code. The pharmacist will break the randomisation code on the request of a researcher; both functionaries are available 24/7.

### Sample size

The primary outcome measure in this study is the percentage of participants completing the protocol in the withdrawal group compared to the percentage in the control group. This will be studied based on a non-inferiority design (the withdrawal group is non-inferior compared to the control group). The sample size is determined using the following formula: n ≥ ((p_c_(1 – p_c_) + p_e_(1 – p_e_)) / δ_0_^2^) x (Z_α_ + Z_β_)^2^ [36, 37]. P_c_ is the percentage completing the protocol in the control group; p_e_ is the percentage completing the protocol in the withdrawal group. A previous study showed that 82% completed the protocol [38]. Because we assume non-inferiority, p_c_ = p_e_ = 0.82. We have assumed a drop-out rate in the control group of 20% (based on previous experiences with research in people with ID). We consider a difference of 20% in the drop-out rate between the two groups to be clinically relevant (δ = 0.2). For a power of 0.8 (Z_β_ = Z_0.2_ = .84), one-tailed with α of 0.025 (Z_α_ = Z_0.025_ = 1.96), we need a sample size of 56 participants in each group (*n* = 112 in total). In order to correct for drop-out from the study that is not related to the intervention, we aim to include 10% more (*n* = 122).

### Outcome measures and diagnostic measurements

#### Outcome measures

The percentage of participants completing the protocol in the withdrawal group compared to the control group is the primary outcome measure. Other outcome measures are differences between the two groups in behavioural changes, newly diagnosed psychiatric disorders, sleep problems and side effects/withdrawal symptoms. Data will be collected according to Table 1.

**Table 1:** Schedule of assessments

Methods / Week number	0	2	4	5	6	8	10	12	13	14	16	21	22	40
Withdrawal group (% AP withdrawal)	**100**	**75**			**50**		**25**			**0**			**0**	^**e**^
Control group (%)	**100**	**100**			**100**		**100**			**100**			**100**	^**e**^
Baseline questionnaire, e.g. participant characteristics	X													
Questionnaires and interviews^a^														
ABC, VAS, CGI, ADAMS, CLE, follow-up questionnaire	X	X	X		X	X	X	X		X	X		X	X
Semi-structured interview	X				X								X	X
Psychiatric disorders^b^														
PAS-ADD	X				X								X	X
Movement disorders^c^														
Rating scales and video registration (SHRS and BARS)	X				X								X	X
Electronic devices for measuring dyskinesia, bradykinesia, akathisia	X				X								X	X
Sleep														
Actiwatch	X	X		X	X				X	X		X		X
Somnography	X											X		
Physical symptoms^d^														
MEDS	X				X								X	X
Heart rate, blood pressure, weight	X	X	X		X	X	X	X		X	X		X	X
Waist circumference	X												X	X
Height	X													
Lab: fasting glucose, triglycerids, cholesterol (total, HDL), cortisol, stored serum	X												X	
CYP2D6, risperidone or pipamperone serum levels	X													
Epilepsy														
Seizure calendar for the last 6 months	X												X	X

^a^ABC, Aberrant Behaviour Checklist (Dutch version AGS); VAS, Visual Analogue Scale; CGI-I, Clinical Global Impression – Improvement scale; ADAMS, Anxiety, Depression and Mood Scale (Dutch version, ADESS); CLE, Checklist Life Events

^b^PAS-ADD, Psychiatric Assessment Schedules for Adults with Developmental Disabilities

^c^SHRS, St. Hans Ratings Scale; BARS, Barnes Akathisia Rating Scale

^d^MEDS, Matson Evaluation of Drug Side effects

^e^follow-up period, own team of physician and behavioural scientist determines further treatment: e.g. maintain the withdrawal, restart AP, maintain AP or start withdrawal if no discontinuation was done

#### Diagnostic measurements

Most assessments in this study will be performed at baseline, and 2 and 4 weeks after each dose reduction. This is to identify signs and differentiate between acute withdrawal symptoms and other symptoms due to a reduced dose of AP that may only be noticed later. The measurements and questionnaires will be repeated during follow-up at 22 weeks (blinded) and 40 weeks (not blinded). The home polysomnography (done in a subgroup), and measurements for diagnosing movement disorders and psychiatric disorders will be performed less frequently to minimise the burden on the participants. If breaking the randomisation code is required e.g. in case of an emergency or severely challenging behaviour, we will ask if it is possible to assess the participant before breaking the randomisation and try to perform all measurements and complete the questionnaires as would normally be done at week 22 (blinded). The week 40 (not blinded) measurements and questionnaires are then rescheduled 18 weeks later.

##### Participant characteristics

Data on participants’ characteristics will be collected using questionnaires which are completed by the physician and behavioural scientist. The following characteristics are collected: sex (male/female), age, level of ID (mild (IQ 50–70), moderate (IQ 35–50), severe (IQ 20–35), profound (IQ < 20)), aetiology of ID (syndrome/acquired brain injury/unknown), autism (present/not present/suspicion) and other psychiatric comorbidities (anxiety disorder/depression/other mood disorder/ attention deficit hyperactivity disorder/attachment disorder), sleep problems (settling problems/night-waking problems/short sleep/sleep-related breathing problems/nocturnal epilepsy/day napping), neurological comorbidity (epilepsy/spasticity/hypotonia), movement disorders (parkinsonism/dyskinesia/dystonia/akathisia/not otherwise specified), and other medical conditions. Details about the living environment, day programme activities, substance abuse, sleeping habits and support for the AP withdrawal will be collected with questionnaires completed by the participant’s professional caregiver. Data will also be collected on the psychotropic drug history for challenging behaviour, other (AP) withdrawal trials, recorded side effects of AP and the Daily Defined Dose (DDD) of the risperidone or pipamperone. Measurements of blood level of risperidone or pipamperone and a CYP2D6 test will be performed at baseline.

##### Behaviour

Behaviour will be measured using the subscales of the Aberrant Behaviour Checklist (ABC; Dutch version), completed by the participant’s main caregiver [39, 40]. The ABC is designed to measure severity of behaviour disorders or treatment effects of psychotropic drugs on challenging behaviour [39, 40]. It consists of 58 items spread over five subscales: irritability, hyperactivity, lethargy, stereotypic behaviour and inappropriate speech. Several studies on the reliability and validity of this instrument have been carried out by the authors as well as by independent researchers [40–46]. The internal consistency of the subscales is excellent for the ‘hyperactivity’ and ‘lethargy’ subscales, good for the ‘irritability’ and ‘stereotypic behaviour’ subscales, and moderate for the ‘inappropriate speech’ subscale. Differences over time in both groups for the five subscales will be compared.

A Visual Analogue Scale (VAS) for behavioural symptoms and the Checklist Life Events (CLE) will be completed by the participant’s main caregiver as well. The VAS (range 0–10) has been adapted for this current study to measure the severity of one or two individualised target behavioural symptoms. The CLE is a checklist for counting life events and is specially designed for the population of adults with ID (good internal consistency (α = 0.81)) [47]. Subjective participant and caregiver opinions and expectations of withdrawal will be assessed with semi-structured interviews. Questionnaires will be given to caregivers, ID physicians and behavioural scientists to investigate their impression of whether the participant is receiving placebo or verum, and to check for any additional interventions during the study. These semi-structured interviews and questionnaires were compiled by our research group, which consists of ID physicians and behavioural scientists. The Clinical Global Impression - Improvement scale (CG-I), which addresses changes in behavioural functioning, will be completed by the behavioural scientist and the physician to measure the severity of the challenging behaviour (range 1–7; or normal, not at all ill - among the most extremely ill) and improvement in the challenging behaviour (range 1–7; or very much improved - very much worse) [48].

To detect underlying existing psychiatric disorders that were not previously identified, the Psychiatric Assessment Schedule for Adults with Developmental Disability (PAS-ADD) clinical interview (Dutch version) will be conducted by the researchers with the participant’s main caregiver and, if possible, with the participant himself/herself [49]. The PAS-ADD clinical interview focuses on axis I diagnoses of the DSM-IV (present/not present) and has been validated for people with ID [49]. In addition, the Anxiety, Depression and Mood Scale (ADAMS; Dutch version) will be completed by professional caregivers to detect depressive and anxiety symptoms [50]. This questionnaire is reliable and valid for adults with ID [50, 51]. The feasibility, test-retest reliability, inter-rater reliability and internal consistency are fair to excellent for this population [50, 51].

##### Sleep

Sleep parameters will be measured using actigraphy (Actiwatch, Philips Respironics: Actiwatch 2 and Actiwatch Spectrum Plus) [52, 53]. Actigraphy is a valid method for determining the sleep-wake rhythm in people with ID [52–56]. Participants wear an Actiwatch (a watch-like devise) for seven consecutive days per measurement to detect changes in sleep parameters and sleep problems, in accordance with the procedure followed by Van de Wouw et al. [21].

To explore AP effects on sleep more extensively, home polysomnography will be performed during two consecutive days in a subgroup of participants who can tolerate this (*n* = 20 in both groups, total *n* = 40). Polysomnography is the gold standard for sleep assessments. This will be performed at baseline and in week 21. The home polysomnography will take place in the home situation to minimise the burden for the participants. In a home polysomnography limited EEG derivations are combined with the registration of respiratory movements (elastic strap around the abdomen and chest), leg movements (patch electrode on the leg), ECG (patch electrodes on the chest) and transcutaneous oxygen saturation measurement on the finger. The air flow is measured by a sensor under the nose. Home polysomnography has proven to be reliable for comfortable outpatient sleep recording [53].

##### Movement disorders

Movement disorders will be assessed using rating scales and new electronic devices. The rating scales have been chosen for their applicability in the population of adults with ID. Earlier research in people with ID recommended using objective instruments for the measurement of akathisia in addition to the rating lists [57]. Where possible, new objective electronic devices have been added to see if it is feasible to measure preclinical movement disorders in this population in this way.

The following rating scales will be used: the St. Hans rating scale (SHRS) and the Barnes Akathisia Rating Scale (BARS) [58–60]. The SHRS has been validated in psychiatric patients and has a high inter-rater reliability [58, 59]. The SHRS has four subscales: dyskinesia (8 body areas), parkinsonism (8 items), akathisia and dystonia (4 body areas), measured on a 7-point rating scale (range 0–6) [58, 59]. The precise interpretation of the SHRS subscores and total scores is not clear [61]. The scores (range 0–6) for the dyskinesia, parkinsonism and dystonia subscales will be used in our study. The BARS is a rating scale for akathisia; it has a good inter-rater reliability in the psychiatric population and has been used in previous research in people with ID [60, 62]. Akathisia will be defined based on objective symptoms (range 0–3), subjective symptoms (range 0–3) and global clinical assessment (range 0–5) [60].

New electronic devices will also be used to measure bradykinesia, dyskinesia and akathisia. Bradykinesia will be assessed using wireless inertial sensors (Mtw, XSENS). In the psychiatric population, these sensors are valid and reliable when compared with the Unified Parkinson’s Disease Rating Scale (UPDRS) bradykinesia subscale (a validated rating scale) [63, 64]. Dyskinesia will be measured using a device that assesses dyskinesia by measuring variability in force while applying pressure to a button. This device can measure dyskinesia objectively and reliably [65, 66]. An actigraph (GT3X+), worn on a belt, will be used to measure akathisia. This instrument has been used in the psychiatric population to measure akathisia [67, 68], but has never been used in a population with ID.

##### Withdrawal symptoms and side effects

Withdrawal symptoms and other side effects will be measured with the Matson Evaluation of Drug Side Effects (MEDS). The MEDS is a 90-item validated assessment that can detect side effects of psychotropic drugs in people with ID [69–71]. It includes nine domains: cardiovascular and hematologic effects, gastrointestinal effects, endocrine/genitourinary effects, eye/ear/nose/throat effects, skin/allergy/temperature effects, CNS (central nervous system)-general, CNS-dystonia, CNS-parkinsonism/dyskinesia and CNS-behavioural-akathisia [69]. Each item is rated as to the occurrence, severity and duration [69]. This assessment has been translated into Dutch (using ‘forward and backward’ translation). Furthermore, a physical examination will be performed repeatedly to measure height, weight, waist circumference, blood pressure and heart rate; see Table 1. Blood tests will be performed to give the glucose and lipid profile and determine any metabolic syndrome. Changes in epileptic seizures will be noted during the study.

### Data collection and management

All the assessments will be carried out by trained behavioural scientists, ID physicians or trainee ID physicians. Each participant will be measured by the same researcher throughout the entire study period wherever possible. The clinical assessment (height, weight, blood pressure etc.) will be performed by a doctor’s assistant or nurse. Data from these assessments will be collected and stored using a web-based electronic data collection system (OpenClinica). Data from the questionnaires and surveys will be collected using Lime Survey and GemsTracker. These programs are password protected.

Data will be processed and stored for a period of 15 years. At the end of the study, signed informed consent forms and other participant documents will be stored at the participant’s care organisation. Recruitment of the participants started in March 2019 and is still ongoing as at July 2021. We expect to complete data collection by summer 2022.

### Statistical analysis

#### Baseline characteristics, short-term and long-term analyses

Descriptive statistics will be used to present the participant characteristics of the two groups at baseline. Baseline imbalances will be investigated using the independent t-test for normally distributed continuous data, the Mann-Whitney test for non-normally distributed continuous data and the chi-square test for categorical data. We will investigate the short-term (intervention period: baseline – week 22) and long-term (including the follow-up period until week 40) effects of AP withdrawal.

#### First hypothesis

There will be a 95% Wilson score interval calculated for p_c_-p_e_. If the upper limit of this interval is below the non-inferiority margin δ of 0.2, we will reject the null hypothesis and we will have shown that the success rate in the AP withdrawal group is not lower than in the control group. As an additional analysis, the time to drop-out will be analysed using a Cox proportional hazard model. Non-inferiority is determined as: the upper limit of the two-tailed 95% confidence interval for the withdrawal group’s hazard ratio with respect to the control group is less than 1.25. We will explore if baseline imbalances impact the results, and adjustments will be made if necessary.

#### Second and third hypotheses

Linear mixed models will be used to evaluate differences between the groups for the sleep, psychiatric and behavioural problems and withdrawal symptoms at the various measurement points. We will use a model in which we assume that the evolution of both groups is the same at baseline and then develops according to a third-degree or smaller polynomial. We will first choose the most suitable correlation structure based on the AIC (Akaike information criterion) and then use the likelihood ratio to test whether we can simplify the structure of the average profiles. We will explore whether baseline imbalances impact the results and adjustments will be made if necessary. Finally, the mean profiles of both groups will be compared with each other using a likelihood ratio test where the null hypothesis is that they are the same over the entire study period.

## Discussion

To our knowledge, this is the first multicentre double-blind, placebo-controlled randomised AP withdrawal trial in people with ID and challenging behaviour aimed at unravelling the mechanisms explaining why off-label AP withdrawal so often fails.

A unique and systematic recruitment method will be used. The aim of this method is to reduce selection bias as much as possible. In previous studies, physicians and/or behavioural scientists identified potential participants and did not always use a clear, properly described method to do so. In our study, we ask the physicians and behavioural scientists to identify potential participants using the medication lists. After a systematic check of the inclusion and exclusion criteria by their physician and behavioural scientist, potential participants will be approached and asked if they would like to receive the study information. If physicians and behavioural scientists decide not to approach potential participants, the reasons why will be noted anonymously. This ensures that physicians and behavioural scientists think systematically about approaching a participant and/or legal representative for participation and it will prevent them from approaching only those participants who they believe are likely to have a successful withdrawal. Also, the reasons why potential participants and/or their legal representatives decide not to participate will be noted.

It is important to understand why AP withdrawal often fails in people with ID and challenging behaviour. The international policy is to reduce the prescription or continuation of AP in people with ID and challenging behaviour [29, 31]. Because withdrawal of AP often fails, care professionals caring for people with ID are calling for guidelines and interventions supporting AP withdrawal. By assessing the mechanisms explaining why AP withdrawal is often not successful, this study will provide important knowledge about AP use and AP withdrawal that is needed for these guidelines. By conducting this study, we will learn more about the influence of attitudes and apprehension on the AP withdrawal outcome. More knowledge will be obtained about the influence of AP on previously undiagnosed psychiatric disorders, on sleep problems and on behavioural problems. The study will also provide more knowledge about the influence of withdrawal symptoms on AP withdrawal failure.

Evidence regarding AP withdrawal, the effects of AP and AP withdrawal will serve as input for the guidelines and may result in a decrease of off-label AP use in people with ID. Because of the highly prevalent and clinically relevant side effects of AP, AP withdrawal and more consideration prior to starting AP use will result in important health benefits for people with ID.

## Data Availability

Generated data or analysed data from the current study are available from the corresponding author on reasonable request.

## Abbreviations

ABC: Aberrant Behaviour Checklist (Dutch version AGS)
ADAMS: Anxiety, Depression and Mood Scale (Dutch version, ADESS)
AP: Antipsychotic(s)
BARS: Barnes Akathisia Rating Scale
CGI-I: Clinical Global Impression – Improvement scale
CLE: Checklist Life Events
DDD: Daily Defined Dose
HA-ID: ‘Healthy Ageing and Intellectual Disabilities’
ID: Intellectual disability
MEDS: Matson Evaluation of Drug Side effects
PAS-ADD: Psychiatric Assessment Schedules for Adults with Developmental Disabilities
SHRS: St. Hans Ratings Scale
UPDRS: Unified Parkinson’s Disease Rating Scale
VAS: Visual Analogue Scale

## References

[1] Levitas AS, Hurley AD (2006). The history behind the use of antipsychotic medications in persons with intellectual disability: part I. Mental Health Aspects Dev Disabil.

[2] de Kuijper G, Hoekstra P, Visser F, Scholte FA, Penning C, Evenhuis H (2010). Use of antipsychotic drugs in individuals with intellectual disability (ID) in the Netherlands: prevalence and reasons for prescription. J Intellect Disabil Res.

[3] Tsiouris JA, Kim SY, Brown WT, Pettinger J, Cohen IL (2013). Prevalence of psychotropic drug use in adults with intellectual disability: positive and negative findings from a large scale study. J Autism Dev Disord.

[4] Hsu SW, Chiang PH, Chang YC, Lin JD, Tung HJ, Chen CY (2014). Trends in the use of psychotropic drugs in people with intellectual disability in Taiwan: a nationwide outpatient service study, 1997-2007. Res Dev Disabil.

[5] Clarke DJ, Kelley S, Thinn K, Corbett JA (1990). Psychotropic drugs and mental retardation: 1. Disabilities and the prescription of drugs for behaviour and for epilepsy in three residential settings. J Ment Defic Res.

[6] Stolker JJ, Koedoot PJ, Heerdink ER, Leufkens HG, Nolen WA (2002). Psychotropic drug use in intellectually disabled group-home residents with behavioural problems. Pharmacopsychiatry..

[7] Branford D (1996). A review of antipsychotic drugs prescribed for people with learning disabilities who live in Leicestershire. J Intellect Disabil Res.

[8] van Schrojenstein Lantman-de Valk HM, Kessels AG, Haveman MJ, Maaskant MA, Urlings HF, van den Akker M (1995). [Drug use by mentally handicapped persons in institutions and family-replacing residential facilities] Medicijngebruik door verstandelijk gehandicapten in instituten en gezinsvervangende tehuizen. Ned Tijdschr Geneeskd.

[9] Emerson E, Kiernan C, Alborz A, Reeves D, Mason H, Swarbrick R, Mason L, Hatton C (2001). The prevalence of challenging behaviors: a total population study. Res Dev Disabil.

[10] Holden B, Gitlesen JP (2006). A total population study of challenging behaviour in the county of Hedmark, Norway: prevalence, and risk markers. Res Dev Disabil.

[11] Deb S, Thomas M, Bright C (2001). Mental disorder in adults with intellectual disability. 2: the rate of behaviour disorders among a community-based population aged between 16 and 64 years. J Intellect Disabil Res.

[12] Jones S, Cooper SA, Smiley E, Allan L, Williamson A, Morrison J (2008). Prevalence of, and factors associated with, problem behaviors in adults with intellectual disabilities. J Nerv Ment Dis.

[13] Dudley JR, Ahlgrim-Delzell L, Calhoun ML (1999). Diverse diagnostic and behavioural patterns amongst people with a dual diagnosis. J Intellect Disabil Res.

[14] de Winter CF, Jansen AA, Evenhuis HM (2011). Physical conditions and challenging behaviour in people with intellectual disability: a systematic review. J Intellect Disabil Res.

[15] Cooper SA, Smiley E, Morrison J, Williamson A, Allan L (2007). Mental ill-health in adults with intellectual disabilities: prevalence and associated factors. Br J Psychiatry.

[16] Hassiotis A, Strydom A, Hall I, Ali A, Lawrence-Smith G, Meltzer H, Head J, Bebbington P (2008). Psychiatric morbidity and social functioning among adults with borderline intelligence living in private households. J Intellect Disabil Res.

[17] Holden B, Gitlesen JP (2003). Prevalence of psychiatric symptoms in adults with mental retardation and challenging behaviour. Res Dev Disabil.

[18] Braam W, van Duinen-Maas MJ, Festen DAM, van Gelderen I, Huisman SA, Tonino MAM (2014). Medische zorg voor patiënten met een verstandelijke beperking: Prelum.

[19] Brylewski J, Duggan L (2004). Antipsychotic medication for challenging behaviour in people with learning disability. Cochrane Database Syst Rev.

[20] Tyrer P, Oliver-Africano PC, Ahmed Z, Bouras N, Cooray S, Deb S, Murphy D, Hare M, Meade M, Reece B, Kramo K, Bhaumik S, Harley D, Regan A, Thomas D, Rao B, North B, Eliahoo J, Karatela S, Soni A, Crawford M (2008). Risperidone, haloperidol, and placebo in the treatment of aggressive challenging behaviour in patients with intellectual disability: a randomised controlled trial. Lancet..

[21] van de Wouw E, Evenhuis HM, Echteld MA (2013). Objective assessment of sleep and sleep problems in older adults with intellectual disabilities. Res Dev Disabil.

[22] Correll CU (2010). From receptor pharmacology to improved outcomes: individualising the selection, dosing, and switching of antipsychotics. Eur Psychiatry.

[23] Brylewski J, Wiggs L (1999). Sleep problems and daytime challenging behaviour in a community-based sample of adults with intellectual disability. J Intellect Disabil Res.

[24] de Kuijper G, Mulder H, Evenhuis H, Scholte F, Visser F, Hoekstra PJ (2013). Determinants of physical health parameters in individuals with intellectual disability who use long-term antipsychotics. Res Dev Disabil.

[25] de Kuijper GM, Mulder H, Evenhuis H, Visser F, Hoekstra PJ (2014). Effects of discontinuation of long-term used antipsychotics on prolactin and bone turnover markers in patients with intellectual disability. J Clin Psychopharmacol.

[26] Fodstad JC, Bamburg JW, Matson JL, Mahan S, Hess JA, Neal D, Holloway J (2010). Tardive dyskinesia and intellectual disability: an examination of demographics and topography in adults with dual diagnosis and atypical antipsychotic use. Res Dev Disabil.

[27] Matson JL, Fodstad JC, Neal D, Dempsey T, Rivet TT (2010). Risk factors for tardive dyskinesia in adults with intellectual disability, comorbid psychopathology, and long-term psychotropic use. Res Dev Disabil.

[28] de Kuijper G, Mulder H, Evenhuis H, Visser F, Hoekstra PJ (2013). Effects of controlled discontinuation of long-term used antipsychotics on weight and metabolic parameters in individuals with intellectual disability. J Clin Psychopharmacol.

[29] Sheehan R, Hassiotis A. Reduction or discontinuation of antipsychotics for challenging behaviour in adults with intellectual disability: a systematic review. Lancet Psychiatry. 2017;4(3):238-56. 10.1016/S2215-0366(16)30191-2 27838214

[30] de Kuijper G, Evenhuis H, Minderaa RB, Hoekstra PJ (2014). Effects of controlled discontinuation of long-term used antipsychotics for behavioural symptoms in individuals with intellectual disability. J Intellect Disabil Res.

[31] Beumer S, Anne Maria Maes-Festen D (2017). Reduction or discontinuation of antipsychotics for challenging behaviour in adults with intellectual disability: why does it fail?. Lancet Psychiatry.

[32] Christian L, Snycerski SM, Singh NN, Poling A (1999). Direct service staff and their perceptions of psychotropic medication in non-institutional settings for people with intellectual disability. J Intellect Disabil Res.

[33] Ahmed Z, Fraser W, Kerr MP, Kiernan C, Emerson E, Robertson J, Felce D, Allen D, Baxter H, Thomas J (2000). Reducing antipsychotic medication in people with a learning disability. Br J Psychiatry.

[34] Cerovecki A, Musil R, Klimke A, Seemuller F, Haen E, Schennach R (2013). Withdrawal symptoms and rebound syndromes associated with switching and discontinuing atypical antipsychotics: theoretical background and practical recommendations. CNS Drugs.

[35] Jesner OS, Aref-Adib M, Coren E (2007). Risperidone for autism spectrum disorder. Cochrane Database Syst Rev.

[36] Zhong B (2009). How to calculate sample size in randomized controlled trial?. J Thorac Dis.

[37] Noordzij M, Tripepi G, Dekker FW, Zoccali C, Tanck MW, Jager KJ (2010). Sample size calculations: basic principles and common pitfalls. Nephrol Dial Transplant.

[38] Ramerman L, de Kuijper G, Scheers T, Vink M, Vrijmoeth P, Hoekstra PJ (2019). Is risperidone effective in reducing challenging behaviours in individuals with intellectual disabilities after 1 year or longer use? A placebo-controlled, randomised, double-blind discontinuation study. J Intellect Disabil Res.

[39] Aman MG, Singh NN, Stewart AW, Field CJ (1985). The aberrant behavior checklist: a behavior rating scale for the assessment of treatment effects. Am J Ment Defic.

[40] Aman MG, Singh NN, Stewart AW, Field CJ (1985). Psychometric characteristics of the aberrant behavior checklist. Am J Ment Defic.

[41] Aman MG, Richmond G, Stewart AW, Bell JC, Kissel RC (1987). The aberrant behavior checklist: factor structure and the effect of subject variables in American and New Zealand facilities. Am J Ment Defic.

[42] Paclawskyj TR, Matson JL, Bamburg JW, Baglio CS (1997). A comparison of the diagnostic assessment for the severely handicapped-II (DASH-II) and the aberrant behavior checklist (ABC). Res Dev Disabil.

[43] Rojahn J, Aman MG, Matson JL, Mayville E (2003). The aberrant behavior checklist and the behavior problems inventory: convergent and divergent validity. Res Dev Disabil.

[44] Rojahn J, Helsel WJ (1991). The aberrant behavior checklist with children and adolescents with dual diagnosis. J Autism Dev Disord.

[45] Rojahn J, Rowe EW, Kasdan S, Moore L, van Ingen DJ (2011). Psychometric properties of the aberrant behavior checklist, the anxiety, depression and mood scale, the assessment of dual diagnosis and the social performance survey schedule in adults with intellectual disabilities. Res Dev Disabil.

[46] Aman MG (2010). Annotated biography on the aberrant behavior Checkllist (ABC). Unpublished manuscript.

[47] Hermans H, Evenhuis HM (2012). Life events and their associations with depression and anxiety in older people with intellectual disabilities: results of the HA-ID study. J Affect Disord.

[48] Forkmann T, Scherer A, Boecker M, Pawelzik M, Jostes R, Gauggel S (2011). The clinical global impression scale and the influence of patient or staff perspective on outcome. BMC Psychiatry.

[49] Moss S, Prosser H, Costello H, Simpson N, Patel P, Rowe S, Turner S, Hatton C (1998). Reliability and validity of the PAS-ADD checklist for detecting psychiatric disorders in adults with intellectual disability. J Intellect Disabil Res.

[50] Hermans H, Jelluma N, van der Pas FH, Evenhuis HM (2012). Feasibility, reliability and validity of the Dutch translation of the anxiety, depression and mood scale in older adults with intellectual disabilities. Res Dev Disabil.

[51] Hamers PCM, van Ool JS, Festen DAM, Hendriksen JGM, Bindels PJE, Hermans H (2019). Reliability and validity of the Dutch anxiety, depression and mood scale in adults aged <50 years with intellectual disabilities. J Appl Res Intellect Disabil.

[52] Pollak CP, Tryon WW, Nagaraja H, Dzwonczyk R (2001). How accurately does wrist actigraphy identify the states of sleep and wakefulness?. Sleep..

[53] Fietze I, Penzel T, Partinen M, Sauter J, Kuchler G, Suvoro A (2015). Actigraphy combined with EEG compared to polysomnography in sleep apnea patients. Physiol Meas.

[54] Morgenthaler T, Alessi C, Friedman L, Owens J, Kapur V, Boehlecke B, Brown T, Chesson A Jr, Coleman J, Lee-Chiong T, Pancer J, Swick TJ, Standards of Practice Committee, American Academy of Sleep Medicine (2007). Practice parameters for the use of actigraphy in the assessment of sleep and sleep disorders: an update for 2007. Sleep..

[55] van Dijk E, Hilgenkamp TI, Evenhuis HM, Echteld MA (2012). Exploring the use of actigraphy to investigate sleep problems in older people with intellectual disability. J Intellect Disabil Res.

[56] Hare DJ, Jones S, Evershed K (2006). Objective investigation of the sleep-wake cycle in adults with intellectual disabilities and autistic spectrum disorders. J Intellect Disabil Res.

[57] Bodfish JW, Newell KM, Sprague RL, Harper VN, Lewis MH (1997). Akathisia in adults with mental retardation: development of the akathisia ratings of movement scale (ARMS). Am J Ment Retard.

[58] Gerlach J, Korsgaard S, Clemmesen P, Lauersen AM, Magelund G, Noring U (1993). The St. Hans rating scale for extrapyramidal syndromes: reliability and validity. Acta Psychiatr Scand.

[59] Van Harten PN (2004). Meetinstrumenten bij motorische bijwerkingen. Tijdschr Psychiatr.

[60] Barnes TR (1989). A rating scale for drug-induced akathisia. Br J Psychiatry.

[61] Knol W, Keijsers CJ, Jansen PA, van Marum RJ (2010). Systematic evaluation of rating scales for drug-induced parkinsonism and recommendations for future research. J Clin Psychopharmacol.

[62] Scheifes A, Walraven S, Stolker JJ, Nijman HL, Tenback DE, Egberts TC, et al. Movement Disorders in Adults With Intellectual Disability and Behavioral Problems Associated With Use of Antipsychotics. J Clin Psychopharmacol. 2016;36(4):308-13. 10.1097/JCP.0000000000000528 27300250

[63] Mentzel TQ, Lieverse R, Levens A, Mentzel CL, Tenback DE, Bakker PR, Daanen HAM, van Harten PN (2016). Reliability and validity of an instrument for the assessment of bradykinesia. Psychiatry Res.

[64] Mentzel TQ, Mentzel CL, Mentzel SV, Lieverse R, Daanen HA, van Harten PN (2016). Instrumental assessment of bradykinesia: a comparison between motor tasks. IEEE J Biomed Health Inform.

[65] Caligiuri MP, Lohr JB (1990). Fine force instability: a quantitative measure of neuroleptic-induced dyskinesia in the hand. J Neuropsychiatr Clin Neurosci.

[66] Caligiuri MP, Lohr JB, Rotrosen J, Adler L, Lavori P, Edson R, Tracy K (1997). Reliability of an instrumental assessment of tardive dyskinesia: results from VA cooperative study #394. Psychopharmacology.

[67] Poyurovsky M, Nave R, Epstein R, Tzischinsky O, Schneidman M, Barnes TR (2000). Actigraphic monitoring (actigraphy) of circadian locomotor activity in schizophrenic patients with acute neuroleptic-induced akathisia. Eur Neuropsychopharmacol.

[68] Gardos G, Teicher MH, Lipinski JF, Matthews JD, Morrison L, Conley C (1992). Quantitative assessment of psychomotor activity in patients with neuroleptic-induced akathisia. Prog Neuro-Psychopharmacol Biol Psychiatry.

[69] Matson JL, Mayville EA, Bielecki J, Barnes WH, Bamburg JW, Baglio CS (1998). Reliability of the Matson evaluation of drug side effects scale (MEDS). Res Dev Disabil.

[70] Matson JL, Cervantes PE (2013). Current status of the Matson evaluation of drug side effects (MEDS). Res Dev Disabil.

[71] Garcia MJ, Matson JL (2008). Akathisia in adults with severe and profound intellectual disability: a psychometric study of the MEDS and ARMS. J Intellect Develop Disabil.

